# Double-Layer Fatty Acid Nanoparticles as a Multiplatform for Diagnostics and Therapy

**DOI:** 10.3390/nano12020205

**Published:** 2022-01-08

**Authors:** María Salvador, José Luis Marqués-Fernández, José Carlos Martínez-García, Dino Fiorani, Paolo Arosio, Matteo Avolio, Francesca Brero, Florica Balanean, Andrea Guerrini, Claudio Sangregorio, Vlad Socoliuc, Ladislau Vekas, Davide Peddis, Montserrat Rivas

**Affiliations:** 1 Department of Physics and IUTA, Campus de Viesques, University of Oviedo, 33203 Gijón, Spain; salvadormaria@uniovi.es (M.S.); UO254204@uniovi.es (J.L.M.-F.); jcmg@uniovi.es (J.C.M.-G.); 2 Institute of Structure of Matter—National Research Council (CNR), Monterotondo Scalo, 00015 Rome, Italy; Dino.Fiorani@ism.cnr.it; 3 Department of Physics, Università degli Studi di Milano and INFN, 20133 Milano, Italy; paolo.arosio@unimi.it; 4 Department of Physics, Università degli Studi di Pavia and INFN, 27100 Pavia, Italy; matteo.avolio01@universitadipavia.it (M.A.); francesca.brero@unipv.it (F.B.); 5 Romanian Academy—Timisoara Branch, Center for Fundamental and Advanced Technical Research, Laboratory for Magnetic Fluids, 300222 Timisoara, Romania; floricabalanean@gmail.com (F.B.); vsocoliuc@gmail.com (V.S.); vekas.ladislau@gmail.com (L.V.); 6 INSTM and Dipartimento di Chimica “Ugo Schiff”, Università di Firenze, 50019 Sesto Fiorentino, Italy; andrea.guerrini@sns.it (A.G.); csangregorio@iccom.cnr.it (C.S.); 7 ICCOM-CNR and INSTM, 50019 Sesto Fiorentino, Italy; 8 Department of Chemistry and Industrial Chemistry, Università degli Studi di Genova, 16146 Genova, Italy

**Keywords:** magnetic nanoparticles, magnetic hyperthermia, magnetic relaxation, magnetic resonance imaging, biosensor, lateral flow immunoassays, inductive sensing

## Abstract

Today, public health is one of the most important challenges in society. Cancer is the leading cause of death, so early diagnosis and localized treatments that minimize side effects are a priority. Magnetic nanoparticles have shown great potential as magnetic resonance imaging contrast agents, detection tags for in vitro biosensing, and mediators of heating in magnetic hyperthermia. One of the critical characteristics of nanoparticles to adjust to the biomedical needs of each application is their polymeric coating. Fatty acid coatings are known to contribute to colloidal stability and good surface crystalline quality. While monolayer coatings make the particles hydrophobic, a fatty acid double-layer renders them hydrophilic, and therefore suitable for use in body fluids. In addition, they provide the particles with functional chemical groups that allow their bioconjugation. This work analyzes three types of self-assembled bilayer fatty acid coatings of superparamagnetic iron oxide nanoparticles: oleic, lauric, and myristic acids. We characterize the particles magnetically and structurally and study their potential for resonance imaging, magnetic hyperthermia, and labeling for biosensing in lateral flow immunoassays. We found that the myristic acid sample reported a large $r_{2}$ relaxivity, superior to existing iron-based commercial agents. For magnetic hyperthermia, a significant specific absorption rate value was obtained for the oleic sample. Finally, the lauric acid sample showed promising results for nanolabeling.

## 1. Introduction

Today more than ever, there is the need to develop tools that help us solve emerging biomedical problems. According to the World Health Organization, cancer ranks as the leading cause of death in every country of the world [1]. Nearly 10 million cancer deaths happened in 2020. The recent coronavirus disease 2019 (COVID-19) pandemic pushed the health systems to their limits. While they were focusing their resources on COVID-19 patients, others suffered from severe delays. In particular, undiagnosed cancers might emerge in the next few years with incremented risk due to these screening gaps [2,3]. Unquestionably, any disease, especially cancer, should be diagnosed and treated without delay: treatments are simpler and more effective at an initial stage.

The public scene is now used to the term “rapid diagnostic tests” thanks to the ongoing COVID-19 pandemic. Most of them are based on lateral flow assays (LFAs), which have carved out a solid reputation thanks to their quickness, portability, easy use, and affordability [4]. These characteristics make them ideal for a point-of-care solution for diagnostic. LFAs rely on paper microfluidics and bio-recognition. The tests consist of a strip of nitrocellulose along which the biological sample flows by capillarity. A recognition molecule previously immobilized across a line in the strip captures the molecule of interest. A yes/no answer (presence/absence) is observed by the naked eye when the biomolecule is labeled with a colored particle or enzyme, as in the home pregnancy test. When it is necessary to quantify the signal, commercial optical readers can be used. However, this method is sensitive to interferences coming from the ambient light or staining from the membrane. Optical methods can detect only the labeling particles on the membrane surface but not those below it [5]. Overcoming these limitations will boost the spread of the technique to more biomedical applications that require quantifying capacity.

Non-invasive diagnosis in vivo by magnetic resonance imaging (MRI) provides good spatial resolution, and real-time imaging with good penetration depth. Despite the excellent quality of the images, there are cases in which contrast is too poor [6]. Exogenous contrast agents selectively shorten the proton relaxation time in the region of interest, and thus, the contrast is enhanced. So far, contrast agents based on gadolinium have been used, but they lack specificity and are toxic [7].

Once the disease is present, less aggressive, more effective, and affordable treatments are needed. Hyperthermia consists of increasing the temperature to induce irreversible damage to the malignant cells. Locally applied hyperthermia is well controlled in terms of heat uniformity and collateral damages to healthy cells and is preferred to extensive treatments [8]. However, only regions close to the surface can be treated this way. In principle, hyperthermia by laser or microwave light only penetrates a small depth into the tissues and can be used to treat superficial tumors. Nanoparticles that serve as hyperthermia mediators by absorbing radiation and heating their surroundings are used to penetrate deeper regions of the body. Additionally, magnetic hyperthermia has been proved as a novel antimicrobial approach. The controlled release of cupric ions from copper nanoparticles, mediated by the magnetic activation of Fe_3_O_4_ nanoparticles, has been recently proposed [9]. Exploiting the synergistic effect of this composite material can be helpful for the development of new antibacterial agents, which have become a worldwide priority.

Nanoparticles have the same size as biological entities of interest, allowing their interaction at the molecular level. Moreover, when these particles are magnetic, new capabilities arise, offering new possibilities in the life science fields [10]. Magnetic nanoparticles (MNPs) can be used as magnetic reporters in biosensing. In LFAs, for example, they provide a quantitative signal from the whole volume and are independent of optical conditions [11,12]. They offer the possibility to magnetically isolate and pre-concentrate the analyte, improving the detection [13,14]. MNPs can be designed to be biocompatible and safe. When reduced below a critical size, their behavior is superparamagnetic and avoids self-aggregation, favoring colloidal stability [15]. This allows their use in vivo for imaging and therapy. In the former, MNPs act as contrast agents thanks to their short magnetic relaxation time [16]. In the latter, MNPs are used for the controlled generation of heat. They can be injected into the body and passively (e.g., by the surface biofunctionalization) or actively (e.g., by an external magnetic gradient) target the zone. When exposed to an alternating magnetic field, their magnetic relaxation increases the temperature up to 40–46 °C, damaging or inducing the apoptosis of the cancer cells [17].

In this work, three magnetite core particles have been synthesized by co-precipitation and coated by three different fatty acids: oleic acid (OA), lauric acid (LA), and myristic acid (MA). In principle, fatty acid coatings promote a high superficial crystalline quality of hydrophobic nanoparticles. Then, coating them with a double layer renders the solution hydrophilic (an aqueous suspension is necessary for bioapplications). Additionally, it provides accessible chemical moieties for bioconjugation. The same OA nanoparticles have been previously tested to detect molecules of interest in real samples for food and health care applications, specifically for the magnetic detection and quantification of histamine in wine and extracellular vesicles in human plasma (as potential colorectal cancer biomarkers) [18,19]. In both cases, magnetic nanoparticles yielded better results than the traditional gold labels for detection and quantification in terms of linearity and detection limit. Additionally, the oleic acid-coated magnetic particles favored a smooth flow of the wine along the LFA membrane with no detectable residuals, avoiding the need for pre-treatment to eliminate complex matrixes.

Within this framework, we have tested these magnetic colloids as nanoheaters, MRI contrast agents, and labels in LFA. These preliminary results show these MNPs are a good multi-purpose platform for their application in diagnostic and therapy.

## 2. Materials and Methods

### 2.1. Chemicals and Reagents

FeSO_4_·7H_2_O (>99%), FeCl_3_ (97%), NH_4_OH, oleic acid (90%), myristic acid (>98%), and lauric acid (>98%) were purchased from Merk Schuchardt OHG (Hehenbrunn, Germany) and used without any further modification.

Neutravidin protein was obtained from Thermo Fischer Scientific (Waltham, MA, USA). 1-ethyl-3-[3-dimethylpropyl] carbodiimide (EDC), bovine serum albumin (BSA), biotin-conjugated bovine serum albumin (BBSA), *n*-hydroxysuccinimide (NHS), 2-(*n*-morpholino) ethanesulfonic acid (MES), and Tween20 were purchased from Sigma-Aldrich (Madrid, Spain). Glass fiber membranes (GFCP001000), used as sample pad and backing cards (HF000MC100), were purchased from Millipore (Darmstadt, Germany). Other materials used were nitrocellulose membranes (UniSart CN95, Sartorius, Spain) and absorbent pads (Whatman, Piscataway, NJ, USA).

### 2.2. Synthesis of the Magnetic Nanoparticles

Three different sets of magnetic nanoparticles were synthesized through a chemical co-precipitation method. This procedure was followed by combining steric and electrostatic stabilization in water by adding one of the three fatty acids: OA, LA, and MA. The resulting samples are named OA@NP, LA@NP, and MA@NP. The whole procedure is described elsewhere [20]. Briefly, two solutions of FeSO_4_ and FeCl_3_ with a ratio of iron ions Fe^3+^/Fe^2+^ = 1.7 were mixed under continuous stirring in atmospheric conditions and heated up to 80 °C. To precipitate the particles, a solution of 25% NH_4_OH was added, and just after the co-precipitation reaction, the surfactant was incorporated in a significant excess to start its chemisorption on the magnetite nanoparticles surface. Finally, the phases were separated, followed by decantation, washing steps with distilled water, and elimination of residual salts to disperse the double layer coated magnetic nanoparticles in a weak solution of NH_4_OH, then purified by magnetic separation or filtration.

### 2.3. Physicochemical and Magnetic Characterization of the Magnetic Nanoparticles

Measurements of the particle size were obtained from TEM images (MET JEOL-2000 EX-II, JEOL USA Inc., Peabody, MA, USA), analyzing around 200 particles, and fitting the distribution to a log-normal curve. X-ray diffraction (XRD) analysis was carried out in Seifert XRD 3000 T/T equipment (Rich. Seifert & Co GmbH & KG., Ahrensburg, Germany) using a Mo emitter (Kα: λ1 = 0.709316 Å and λ2 = 0.713607 Å). As a standard, we used Iron (II; III) oxide Puratronic^®^ CAS 1317-61-9 from Alfa Aesar. Thermogravimetric Analysis (TGA) was performed in a 20 µL sample using a Mettler-Toledo TGA/SDTA851 (Mettler-Toledo S.A.E., Barcelona, Spain) in the temperature range from 25 °C to 990 °C, with the heating rate of 10 °C/min, under standard atmosphere.

Fourier-transform infrared spectroscopy (FTIR) was performed in liquid, using an FTIR spectrometer (Agilent Technologies, Spain) coupled with the Varian 620-IR image recorder. Hydrodynamic diameter and ζ-Potential were obtained using dynamic light scattering (DLS) in freshly prepared samples with a Zetasizer Nano ZS ZEN3600 (Malvern Instruments, Malvern, UK) equipped with a solid-state He–Ne laser.

DC-magnetization measurements were performed using a Quantum Design SQUID magnetometer (Quantum Design, San Diego, CA, USA) equipped with a superconducting magnet producing fields up to ±5 T. Initial magnetic susceptibility at room temperature from 1 Hz to 10,000 Hz was measured by a Quantum Design PPMS magnetometer (Quantum Design, San Diego, CA, USA) equipped with a superconducting coil. The magnetization values refer to the mass of iron oxides derived from the mass per volume obtained by the TGA analysis.

### 2.4. Magnetic Hyperthermia Measurements

The evaluation of specific absorption rate (SAR) was performed through calorimetric measurements by recording temperature kinetics of water suspension of MNPs under exposition to an alternating magnetic field (AMF). Measurements were performed using an in-house assembled system comprising a commercial 6 kW Fives Celes power supply, a water-cooled induction coil and a series of variable capacitors for setting the required frequency (183 kHz and 17 kA/m in this case). The amplitude of the magnetic field was assessed using an AMF Life Systems high frequency probe (AMF Life Systems, Auburn Hills, MI, USA). Measurements of the sample temperature were performed by an optical fiber thermometer connected to a digital temperature recorder (Fotemp, OPTOcon GmbH Optical Sensors & Measuring Systems, Dresden, Germany). The SAR was evaluated using the following expression:

(1) $$\mathrm{SAR}=\frac{m_{\mathrm{np}}C_{\mathrm{np}}+m_{s}C_{s}}{m_{\mathrm{np}}}{\left|\frac{\Delta T}{\Delta t}\right|}_{t\approx 0}$$

where $m_{\mathrm{np}}$, $m_{s}$ and $C_{\mathrm{np}}$, $C_{s}$ correspond to the mass and specific heat capacities of the magnetic particles and the solvent and the slope of the temperature; ΔT is the temperature increase in the interval of time Δt of field application. Since the measurements were carried in non-adiabatic conditions, the ΔT/Δt values were extrapolated for t ≈ 0 from the temperature kinetic curves (initial slope value).

### 2.5. Relaxometric Characterization

^1^H nuclear magnetic resonance (NMR) relaxometric measurements were performed at room temperature by measuring the longitudinal $T_{1}$ and transverse $T_{2}$ nuclear relaxation times at the frequencies ν = 56.7 MHz, 21.3 MHz, and 8.5 MHz. The NMR signal detection and generation were obtained by Stelar Spinmaster Fourier transform-nuclear magnetic resonance (FT-NMR) spectrometer (Stelar, Italy). Saturation Recovery (SR) and Carr Purcell Meiboom Gill (CPMG) pulse sequences were used for $T_{1}$ and $T_{2}$ measurements, respectively.

### 2.6. Biofunctionalization of the Magnetic Nanoparticles

**Figure 1:**
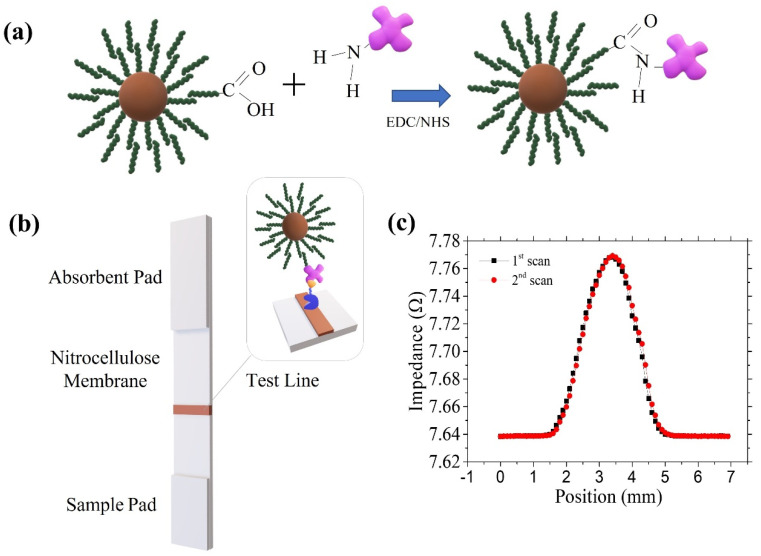
(**a**) Scheme of the particles’ biofunctionalization process with neutravidin by the EDC/NHS chemistry. For simplification, only one functional group has been represented on the surface of the MNPs and the neutravidin. (**b**) Schematic view of a lateral flow strip and its test line: a neutravidin-MNP complex captured by a molecule of biotin. (**c**) Impedance variation for two scans of the OA@NP sample.

The MNPs were conjugated to neutravidin. We used the carboxylic groups present in the fatty acids’ outer layer to link the neutravidin via the carbodiimide chemistry (see Figure 1a). For that purpose, 10 µL of the particles were mixed with 100 µL of EDC (5 mg/mL, MES 50 mM, pH 6.00) and 100 µL of NHS (5 mg/mL, MES pH 6.00), both freshly prepared, and stirred for 10 min. Then, 100 µL of a neutravidin solution with different concentrations (0.75, 1, and 2 mg/mL) was added and shaken for 4 h. To block the residual carboxyl groups on the particle surface, we added 100 µL of the blocking solution (1% BSA, PBS 10 mM, pH 7.4) for 30 min while agitating. The samples were then centrifuged at 14,600 rpm for 20 min, and 300 µL of the supernatant was removed. The pellets were resuspended in fresh PBS 10 mM, pH 7.4. We used DLS measurements to monitor the neutravidin–MNP conjugation.

### 2.7. Lateral Flow Assays

To capture the neutravidin–MNP conjugate in the LFA strip, a test line of biotin has been immobilized across the lateral flow membrane. For this purpose, a 1 mg/mL BBSA solution has been automatically dispensed at a rate of 0.1 µL/mm and then left to dry at room temperature. The sample and absorbent pads were placed onto the plastic adhesive backing card with an overlap of 2 mm. Then, single 5 mm width LFA strips were cut off with a guillotine.

Biotin–neutravidin is widely known in biochemistry for its strength, high thermal and chemical stability, and low non-specific binding. Many biotinylated antibodies are used in lateral flow or other types of immunoassays. For this reason, we chose the neutravidin–biotin paradigm in our experiment to mimic the LFA process and assess the performance of the MNPs as detection nanolabels.

To run the test, 20 µL of the neutravidin-biofuntionalized MNPs were mixed with 80 µL of freshly prepared running buffer (RB) containing 10 mg/mL BSA, 0.5% (*w/v*) Tween20 in PBS (10 mM, pH 7.4). The tests were then carried out in dipstick format by introducing the LFA strip’s sample pad in the microtube, allowing it to flow and dry (see Figure 1b).

### 2.8. Scanning Magneto-Inductive Sensor Measurements

An inductive sensor specifically developed to read out the signal from LFAs was used to evaluate the samples [21]. Its sensing area consists of a planar micro-coil printed on an insulating substrate. The coil is fed with a low amplitude radiofrequency current while its impedance is continuously monitored by a precision four-point auto-balancing impedance analyzer (Agilent 4294A, Keysight Technologies, Santa Rosa, CA, USA) using 16,048G test leads, 500 mV, and 20–110 MHz excitation voltage. For quantification purposes, we slide the sample in smooth contact over the planar coil using a micro-positioner. The coil picks up the varying magnetic flux produced by the magnetic particles on top of it. The electromagnetic induction produces a change in the electric impedance directly proportional to the frequency of the driving current, the particle initial magnetic permeability, and the total volume of their magnetic cores.

The impedance variation during the scanning in 100 micrometers steps (see Figure 1c) is integrated by a trapezoidal method to account for all the MNPs in the sample. The measurement result is given in units of Ω·mm.

The fatty acid-MNPs have been evaluated at two different stages: previously to their bioconjugation and used in the immunoassays.

Firstly, for evaluating the bare fatty acid-MNPs, 10 µL of the three ferrofluids were deposited onto 10 mm × 2 mm blotting papers and left to dry for at least 12 h. The linear correlation between the change of impedance and particle mass was checked for a fixed frequency and type of particles. To facilitate the comparison of the results, we have calculated the sensor sensitivity (Σ) following the use of magnetoimpedance sensors; Σ is defined as the percentage relative impedance variation per unit particle mass m with (Z) and without ($Z_{0}$) MNPs:

(2) $$\Sigma =\frac{1}{m}\frac{Z-Z_{0}}{Z_{0}}\%$$

Secondly, the complexes neutravidin-MNPs were tested as detection labels in LFAs. The biotin test line retains the complexes, and the sensor detects the MNPs in them. In this case, the quantifying result relies not exclusively on the particles’ properties but very significantly on the bioconjugation process efficiency.

## 3. Results and Discussion 

### 3.1. Physicochemical Characterization

**Figure 2:**
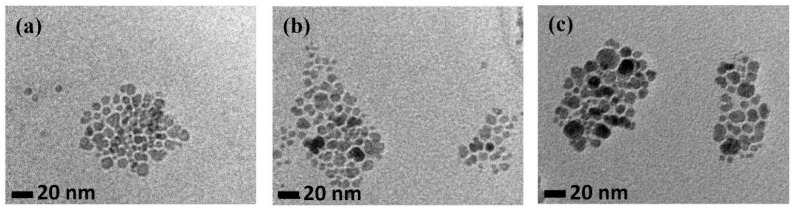
TEM images for samples (**a**) OA@NP, (**b**) LA@NP, and (**c**) MA@NP.

The images obtained by TEM showed a quasi-spherical shape for all the fatty acid-MNPs, as observed in Figure 2. The particle size distribution was obtained by measuring about 200 nanoparticles in various TEM images using ImageJ software. Then, the data were fitted by a log-normal function [22] to obtain the mean particle diameter $d_{\mathrm{TEM}}$, and the standard deviation *σ* (see Table 1). Our results agree with previous work on particles obtained with the same protocol, which bespeaks for the reproducibility of the synthesis method [23]. XRD patterns (see Supplementary Information Figure S1) of the three particle types are in good concordance with the magnetite standard. The broad shape of the diffraction peaks is a clear sign of their nanostructured character. We have used Rietveld refinement and Scherrer equation to calculate the crystallite size presented in Table 1 as $d_{\mathrm{XRD}}$. It is known that $d_{\mathrm{XRD}}$ is an effective size that is smaller than the physical one due to the particle’s non-sphericity. Our results agree with the commonly accepted relation dXRD=34dTEM [24].

We have used the Langevin–Chantrell function [25] to fit our magnetization curves and calculate the magnetic diameter $d_{\mathrm{MAG}}$. All the values obtained for the three samples are slightly smaller than the $d_{\mathrm{TEM}}$, indicating that there is a superficial layer where spin canting and other phenomena may lead to the disorder of the magnetic moments [26]. This dead layer does not seem to be greatly influenced by the coating since it has a very similar value in all of them.

The hydrodynamic diameter $d_{\mathrm{DLS}}$ and the ζ-potential were obtained by DLS and collected in Table 1. The ζ-potential values are negative, and their absolute value is larger than 40 mV, indicating that excellent stability is possible thanks to electrostatic repulsion, which inhibits the particles agglomeration and settling [27]. However, it is worth noticing that all the samples exhibited a certain degree of agglomeration reflected in the high $d_{\mathrm{DLS}}$ values when compared to $d_{\mathrm{TEM}}$. The OA and MA-stabilized ferrofluids present the largest agglomerates. This agglomeration can be seen in Figure 2. Additionally, MA@NP has two mean sizes: 9.5 nm and 25 nm in equal proportions. Agglomeration is a concern in most MNPs studies and the results are not fully conclusive. However, depending on the bioapplication, it can be beneficial or detrimental. Regarding biodetection, it can be advantageous. Stable agglomerated nanoparticles of controlled size provide an amplification of the signal per biomolecule, increasing the detection sensitivity [28]. Furthermore, for magnetic hyperthermia, a slight controlled agglomeration of the nanoheaters can increase the SAR [29].

We performed TGA measurements to elucidate the superficial compounds, their amount, and the magnetic core concentration (see Supplementary Information Figure S2). Most of the weight loss (83% for OA@NP, 81% for LA@NP, and 68% for MA@NP) occurred from 50 °C to 135 °C, attributed to the evaporation of the water and the solvation layer. Another two significant weight losses can be distinguished at 420 °C. These peaks agree with the desorption, decomposition, and evaporation of the surfactant molecules. Specifically, the first weight loss, from 190 °C to 290 °C, suggests the release and decomposition of slightly bound or physically adsorbed surfactant molecules. On the contrary, the second weight loss at higher temperatures (from 290 °C to 420 °C) can be caused by the break of stronger bonds, i.e., chemically bound surfactant molecules decomposition. This double peak pattern in the weight loss curve of fatty acid stabilized nanoparticles confirms the double surfactant layer [30,31,32].

The double layer of fatty acids aims to provide water dispersion and provide superficial carboxylic acid groups that enable the bioconjugation of proteins. Supplementary Information Figure S3 contains the FTIR spectra. They all show the beginning of a steep peak at about 598 cm^−1^ due to the Fe–O bonds’ vibration of the particles’ iron oxide core [33]. The carboxylic acid groups’ vibration is evident in the peak at around 1710 cm^−1^ [34]. Regarding an OA double-layer, Yang et al. studied the differences in the FTIR spectra of single and double layer OA-stabilized nanoparticles [35]. They found that only the peak at 1709 cm^−1^ was present, corresponding to the C–O stretching vibration for free COOH (COO^–-^), which endorses the bilayer formation. This peak exists in the three samples; however, it is better defined in OA@NP and LA@NP but almost imperceptible in MA@NP. Previous studies on similar samples [23,36] pointed out the MA coating’s lower effectiveness when compared to OA and LA. If the coating is incomplete, these residual groups at the MNP surface could be too scarce, and hence, the peak almost negligible. The normal vibration of methyl groups (–CH_2_), due to their symmetric bending, is at 1465 cm^−1^. However, it has been reported that when these alkyl chains are closely packed, this peak splits into two, and the second one shifts to lower wavenumbers. This happens in all our samples and more significantly in OA@NP; in the latter, the bigger organic chains and their double bond bending produce a denser package compared to LA@NP and MA@NP [37].

At the right side of the spectra, all samples showed two characteristic bands at 2920 cm^−1^ and 2850 cm^−1^ due to the symmetric and asymmetric stretching, and the scissoring of C–H bonds of aliphatic compounds, which, mainly in LA@NP and MA@NP, are superimposed to the peak signal of the O–H stretch (usually present at 3300–2500 cm^−1^). Finally, the C–O stretch appeared in the region from 1320 cm^−1^ to 1210 cm^−1^. In summary, the FTIR spectra of OA@NP, LA@NP, and MA@NP confirmed the double layer of fatty acids on the nanoparticle surface and the carboxylic groups.

### 3.2. Magnetic Properties

**Figure 3:**
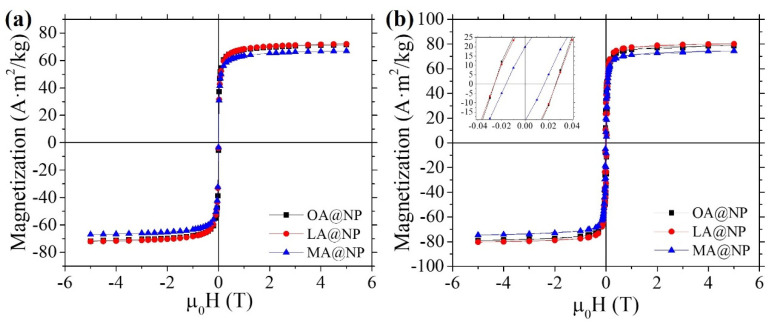
Magnetization curves at (**a**) 250 K and (**b**) 5 K for the three samples OA @NP, LA @NP, and MA@NP. The reduced remanence ($M_{S}/M_{R}$) at 5 K (see Table 2) is far from the theoretical value of 0.5 for non-interacting uniaxial single-domain particles, confirming the presence of non-negligible interparticle interactions, especially for MA@NP.

Magnetization curves at 5 K show no hysteresis at 298 K, while at 5 K there is magnetic hysteresis in all three samples (see Figure 3a,b). The saturation magnetization $M_{S}$ values, calculated by fitting the experimental data to the law of approach to saturation [38], are gathered in Table 2. All the samples show very similar values, close to the value of bulk magnetite (90 Am^2^/kg) [39].

**Figure 4:**
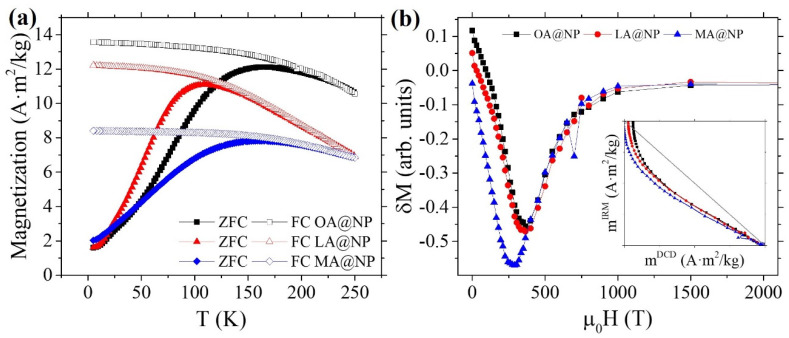
(**a**) ZFC–FC curves of the three samples. (**b**) δM curves calculated from $m^{\mathrm{IRM}}$ and $m^{\mathrm{DCD}}$ curves measured at 5 K. The inset graph shows the Henkel Plots for the three samples OA@NP, LA@NP, and MA@NP.

To assess the particles’ superparamagnetic behavior, we recorded the ZFC–FC curves (see Figure 4). For monodisperse populations with homogeneous magnetic anisotropy, ZFC and FC curves should coincide above the blocking temperature $T_{B}$ at which the particles transit from blocked to superparamagnetic regime [40]. For a polydisperse particle sample, in the absence of interparticle interactions, the difference between the temperature of the maximum $T_{\mathrm{MAX}}$ and the temperature at which both curves merge $T_{\mathrm{IRR}}$, is related to the width of the blocking temperature distribution, and hence, to the particle size distribution. On the other hand, interparticle interactions are present in our samples, as clearly shown by the tendency to flatten the FC curves below $T_{\mathrm{MAX}}$. In such a case, $T_{\mathrm{MAX}}$ is also dependent on the strength of the interparticle interactions.

To further investigate the interaction among particles, we measured the remanent magnetization curves ($m^{\mathrm{IRM}}$ and $m^{\mathrm{DCD}}$) at 5 K with a maximum field of 5 T (see a detailed definition in Supplementary Information Figure S4). For an assembly of non-interacting single-domain particles with uniaxial anisotropy and coherent rotation magnetization process, the two remanence curves accomplish Wohlfarth’s equation [41]:

(3) $$m^{\mathrm{DCD}}\left(H\right)=1-2m^{\mathrm{IRM}}\left(H\right)$$

where $m^{\mathrm{DCD}}\left(H\right)$ and $m^{\mathrm{IRM}}\left(H\right)$ represent the reduced terms $M^{\mathrm{DCD}}\left(H\right)/M_{S}^{\mathrm{DCD}\;}$ and $M^{\mathrm{IRM}}\left(H\right)/M_{S}^{\mathrm{IRM}\;}$, and $M_{S}^{\mathrm{DCD}\;}$ and $M_{S}^{\mathrm{IRM}}$ are the remanence saturation values for the DCD and IRM curves, respectively. The interparticle interactions are confirmed by the $m^{\mathrm{DCD}}\left(H\right)$ versus the $m^{\mathrm{IRM}}\left(H\right)$ curve shape in the Henkel plot (see inset Figure 4b). All the samples lie below the Wohlfarth diagonal line, indicating the predominance of demagnetizing interactions (negative dipolar interactions), i.e., interactions that have the effect of stabilizing the demagnetized state. In contrast, a curve in the region above the Wohlfarth line represents interactions promoting the magnetized state (exchange interactions). δM (see Figure 4b) is defined as:

(4) $$\delta M=m^{\mathrm{DCD}}\left(H\right)-\left[1-2m^{\mathrm{IRM}}\left(H\right)\right]$$

Its dependence on the applied field H allows us to give a quantitative description of the deviations from Wohlfarth Equation (3). MA@NP has the most intense demagnetizing interactions, probably due to its largest particles, with larger magnetic moments, and agglomerates.

### 3.3. Magnetic Hyperthermia Measurements

**Figure 5:**
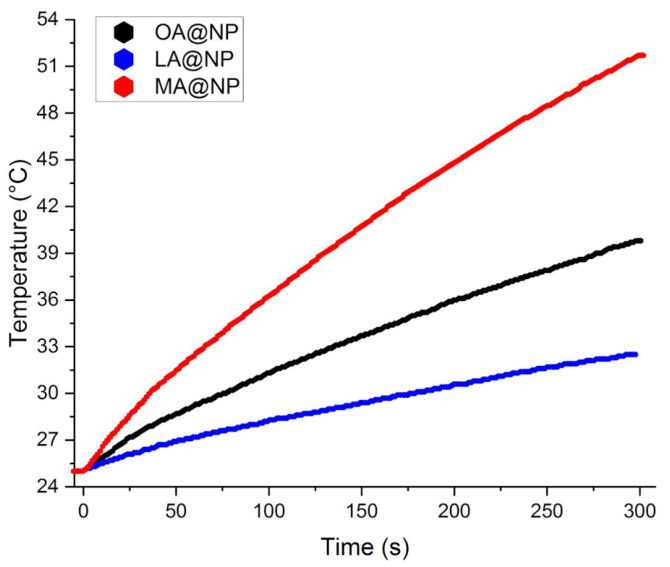
Temperature kinetics of water suspension of OA@NP (conc. 12.0 mg/mL), LA@NP (13.6 mg/mL), and MA@NP (27.5 mg/mL) exposed to an alternating field (17 kA/m amplitude and 183 kHz frequency).

The hyperthermic efficiency of the coated nanoparticles was estimated by recording the temperature increase under the application of an alternating magnetic field of 183 kHz frequency and 17 kA/m amplitude (see Figure 5), which is below the safety threshold for clinical application [42]. The three samples display a high SAR of 29.3 W/g, 12.5 W/g, and 23.8 W/g for OA@NP, LA@NP, and MA@NP, respectively. The observed SAR values scale well with the physical and magnetic diameters of the inorganic core, and their magnetic properties. A larger magnetic moment $\mu =\pi /6d_{MAG}^{3}M_{S}$ indeed corresponds to a higher hyperthermic efficiency. In this respect, OA@NP exhibit the larger magnetic volume compared to the other two samples and similar saturation magnetization. It is worth noting that despite the small average size of the nanoparticles, their intrinsic loss power $\mathrm{ILP}=\mathrm{SAR}\cdot H_{0}^{-2}\cdot f^{-1}$ values, 0.55 nH·m^2^·kg^−1^, 0.24 nH·m^2^·kg^−1^, and 0.45 nH·m^2^·kg^−1^ for OA@NP, LA@NP, and MA@NP, respectively, are remarkably high [43,44]. Interparticle interactions have been found to be either positive or negative, depending on whether crystallographic alignment of the nanoparticles in the aggregate occurs or not [29,45,46,47]. In our case DLS data clearly indicates the presence of agglomerates of large sizes in the OA and MA-stabilized ferrofluids. Thus, although we have no clues on the way the nanoparticles are arranged in the clusters, we can reasonably hypothesize aggregation is the driving force that boosts the heat release capability of our nanosystems.

### 3.4. Relaxometric Characterization

In the framework of biomedical applications, since compounds based on magnetite nanoparticles have been reported as good MRI contrast agents [16], we performed NMR relaxometry studies to evaluate the ability of the fatty-acid-coated MNPs to act, as well as MRI agents.

The measurements of the nuclear relaxivities are suitable to predict the efficiency of magnetic nanoparticles in contrasting MR images [48], given that the nuclear relaxivities, both longitudinal, $r_{1}$, and transverse, $r_{2}$, are defined as:

(5) ri=1Ti,meas−1Ti, diac                i=1,2

where 1Ti,meas is the value measured for the sample with the iron concentration c (mmol/L) and 1Ti, dia represents the nuclear relaxation rate of the diamagnetic host solution (water in our case).

In detail, we performed ^1^H-NMR measurements at frequencies of 56.7 MHz, 21.3 MHz, and 9.0 MHz, respectively, that cover most of the typical fields, H = 1.33 T (near H = 1.5 T), 0.5 T, and 0.2 T, respectively, of MRI tomographs, used both in clinics and in research laboratories.

At these fields, the $r_{1}$ ^1^H-NMR longitudinal nuclear relaxivity values of fatty acid MNPs (reported in Table 3) were a little bit lower or comparable, depending on the MNPs and on the field, to those of Endorem^®^ [49], the withdrawn commercial $T_{2}$ contrast agent still generally used for comparison in literature.

However, in the same clinical fields, the $r_{2}$ ^1^H-NMR transverse nuclear relaxivity values of all fatty acid MNPs (see Table 3) showed on average a 100% increase compared to Endorem^®^, envisaging a potential use of these systems as $T_{2}$ contrast agents in MRI, in particular for MA@NP that showed the highest values. Size and $M_{S}$ are the fundamental parameters for a good $r_{2}$ relaxivity. The broad size distribution of the MA@NP sample will favor the larger $r_{2}$ with respect to Endorem^®^ [6].

Moreover, the higher $r_{2}$ values obtained in our system concerning Endorem^®^ allow us to conclude that, with the same amount of contrast agent, in principle, the contrast-to-noise-ratio in magnetic resonance images might reduce the injected doses.

### 3.5. Biosensing Application

The particles’ applicability as detection labels in inductive sensing with low amplitude magnetic field depends mainly on their initial magnetic susceptibility (for detection and quantification) and their clustering (for signal amplification).

Our inductive sensor has been described elsewhere [50]. It consists of a planar coil whose radio frequency impedance is monitored while the LFA strip is displaced on it in smooth contact in 100 μm steps. In the presence of a magnetic material, the coil acts both as exciting and detecting probe, producing an increase in the impedance proportional to the initial magnetic susceptibility and the number of particles. For monodomain magnetic nanoparticles the susceptibility is maximum at the critical superparamagnetic size (following Néel relaxation model, the initial susceptibility is proportional to the volume as far as the particles are small enough to be superparamagnetic) [51].

Initial magnetic susceptibility ($\chi_{\mathrm{DC}}$) spectra were obtained for the three particles in the range of 10–10,000 Hz, showing no significant variation. Table 2 gathers the $\chi_{\mathrm{DC}}$ values at 10 Hz, which agree with Nèel’s model for a MNPs ensemble with randomly oriented easy axes:

(6) $$\chi_{\mathrm{DC}}=\frac{2}{3}\left(\frac{\mu_{0}\rho^{2}M_{S}^{2}V}{k_{B}T}+\frac{\mu_{0}\rho^{2}M_{S}^{2}}{K_{\mathrm{eff}}}\right)$$

In Equation (6), $\mu_{0}$ is the vacuum permeability, ρ the magnetite density (5170 kg·m^−3^), V the particle mean volume, T the temperature, and $k_{B}$ the Boltzmann constant. Using the parameters of Table 2, and the sizes obtained by TEM, Equation (6) yields $\chi_{\mathrm{DC}}$ values of 21, 13, and 8 for OA@NP, LA@NP, and MA@NP, respectively.

**Figure 6:**
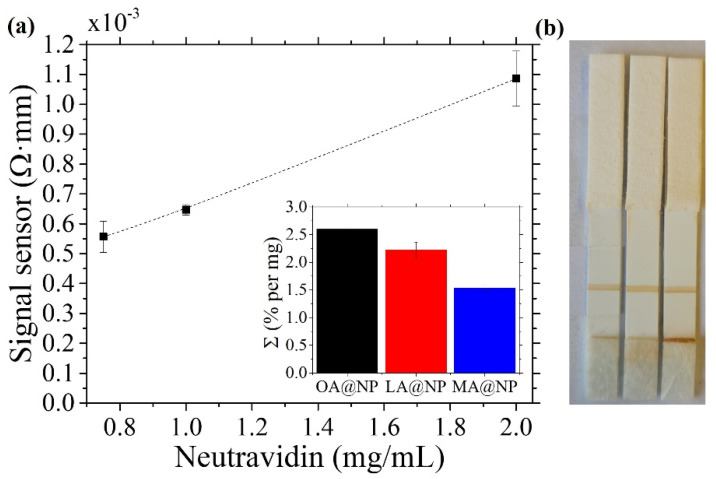
(**a**) Magnetic signal obtained in the sensor for the LFA with neutravidin-conjugated LA@NP particles. The error bars show the standard deviation. The dashed lines serve as a guide to the eye. Inset: Percentage increase per mg of the magnetic signal in the sensor for the three samples. (**b**) Image of the 1 mg/mL of neutravidin LFA run with, from left to right, OA@NP, LA@NP, and MA@NP.

To evaluate the MNPs effect on the sensor signal, we prepared samples depositing original solution droplets on a blotting paper (2 mm × 10 mm size) as described in Section 2.8. The sensitivity for each ferrofluid is shown in the inset of Figure 6a, being 2.60%, 2.22%, and 1.54% per mg for OA@NP, LA@NP, and MA@NP, respectively. Their relative values match the corresponding initial susceptibility results given in Table 2. As expected, in an inductive sensor that uses a very low amplitude exciting field, the particles’ signal should increase with their initial magnetic susceptibility.

Once we assessed the particle performance in the sensor, the next step involved studying the bioconjugation reaction’s influence. The procedure implies the attachment of a biomolecule to the particle. It is performed via the superficial modification of the MNPs, involving the activation of the carboxylic groups at the fatty acid’s outer shell (see Figure 1a,b). During these chemical reactions, some agglomeration of the particles may occur, affecting the number of particles that attach to each protein and, consequently, the signal of the magnetic LFA. The successful functionalization of the nanoparticles with neutravidin was confirmed by testing them against the biotin printed across the membrane as the test line. Streptavidin molecules have four binding sites with an extraordinary affinity for biotin [52,53]. Therefore, when neutravidin is on the nanoparticle surface, the complex formed will become trapped in the biotin line. In Figure 6b, these biotin lines are shown for the three samples when they are bioconjugated with 1 mg/mL of neutravidin. The bioconjugation has been monitored by the $d_{\mathrm{DLS}}$ measurements before and after the process.

In this work, we used neutravidin–biotin affinity to study the three particle types as labels in the LFAs. For this purpose, we have bioconjugated them using three different concentrations of neutravidin. Table 4 displays the parameters of the volume–fraction distributions of sizes obtained from DLS measurements. Although, the DLS technique directly provides intensity-weighted distributions of size, this type of representation is not the best choice for our application because the larger particles become overrepresented (the scattered light is proportional to the 6th power of the particle size) [54]. Transforming into another type of distribution relies on acceptable assumptions such as the sphericity of the particles. Number-weighted distributions are suitable to compare with TEM, but in this study, the most influential parameter is the volume of magnetic material, which is directly proportional to the magnetic moment and, correspondingly, to the sensor detected signal. For this reason, we present in Table 4 the volume fractions of the two-peaked size distributions. Supplementary Information Figure S4 contains additional information on DLS measurements.

Table 4 shows the hydrodynamic sizes before the biofunctionalization in the row labeled as zero-neutravidin content. Typically, hydrodynamic sizes are 10–15% larger than TEM sizes due to the diffuse layer and surfactant at the particle surface. The values for OA@NP and MA@NP indicate that in them, there is already some particle agglomeration before the neutravidin addition.

The agglomerate sizes significantly increase with increasing amounts of protein added, proving the successful conjugation of neutravidin to the particle surface. Some agglomeration can be beneficial for a detection label because it increases the particle:molecule ratio. However, agglomerates larger than 200 nm can be detrimental to the fluid sample flow through the membrane pores and can hamper the antigen–antibody reaction due to steric impediments. These characteristics, the agglomerate size, and abondance, are decisive in applying the particles for LFA.

**Figure 7:**
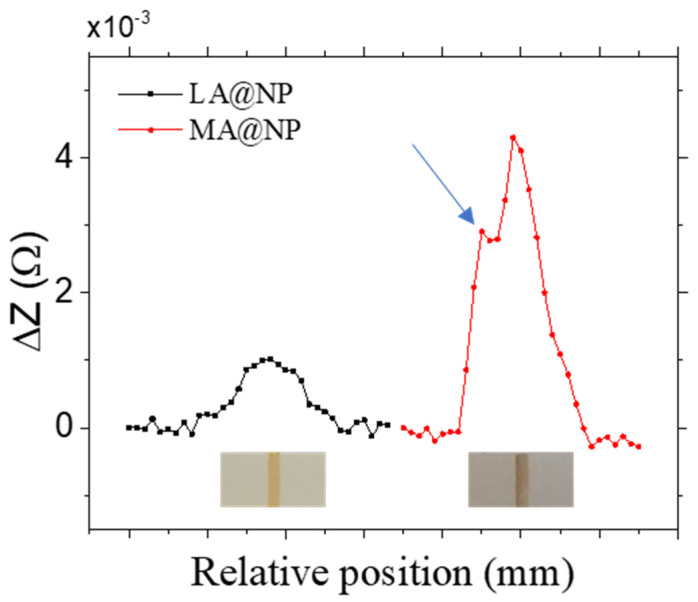
Test line sensor evaluation for the LFAs using LA@NP (black) and MA@NP (red) with 2 mg/mL neutravidin. The horizontal ticks represent 1 mm displacement steps. Bottom: Photographs of both LFAs. The blue arrow points at the peak corresponding to the particle accumulation in the LA@NP.

The DLS-size before bioconjugation in sample LA@NP is 23 nm. Considering the size observed by TEM plus an increase of 10–15%, typical of the diffuse layer and surfactant at the particle surface, the individual DLS-particle size should be around 10 nm. The measured value indicates the existence of clusters formed by 2–5 individual nanoparticles. After bioconjugation, the cluster size increases to approximately 40 nm due to the protein. Only a few larger agglomerates (less than 1% of the total number) with sizes around 150 nm, appear at this stage. They can only be detected in the volume–weighted distributions, as can be seen in Figure S4. The dominating agglomerate size flows through the membrane pores, and the detected signal presents an only peak as expected when the particles distribute themselves homogeneously at the test line. Figure 7 shows the scanning of the LFA with LA@NP sample and 2 mg/mL of neutravidin.

The biofunctionalization of MA@NP produces oversized agglomerates (larger than 200 nm) that correspond to more than 75% of the sample volume, even with the lowest neutravidin concentration. This can be caused by an insufficient MA coating, leading to a higher proportion among the EDC/NHS reagents and the available carboxylic groups during the biofunctionalization. Hence, more of the latter are activated in the process, favoring the agglomeration through the neutravidin. These enormous agglomerates have two different and undesirable effects on the LFAs. The first one is the difficulty of the sample to flow through the membrane pores. It can be seen in the LFAs as a brownish line just after the conjugate pad, as the biggest particles cannot flow and hinder the smaller ones (see Figure 6b). The second effect happens at the test line. Here, the MNPs functionalized with the neutravidin become trapped by the biotin. However, some large neutravidin-MNPs agglomerates form a shield when facing the test line, hindering the rest of the particles’ regular flow, whether functionalized or not. Figure 7 shows the LFA scanning with MA@NP and 2 mg/mL of neutravidin. A darker and thin line can be appreciated and associated with an additional peak in the detected signal. In this case, the larger signal observed for the MA@NP LFA is not due to better detection but to the unspecific accumulation of MNPs.

For sample OA@NP, this behavior is also patent for a neutravidin concentration of 1 mg/mL, and hence, the only valid LFA is the one with the lowest concentration, 0.75 mg/mL (for this concentration, the number of large particles is small and not evident in the number-weighted size distribution). In this case, the excessive agglomeration is likely to be caused by cross-linking among the preexisting agglomerates.

This work has determined that the LA-coating yields the best particles in the study for detection nanolabeling in LFAs. They have superparamagnetic behavior at room temperature, which provides a high initial magnetic susceptibility and contributes to excellent colloidal stability, surface carboxylic groups before bioconjugation, and an initial beneficial monodisperse agglomeration, which increases the magnetic material per unit molecule without hindering the regular flowing.

## 4. Conclusions

Magnetic nanoparticles are a driving force in current solutions for medical problems. Their properties can make them an ideal multiplatform for fast, safe, and reliable diagnosis and therapy. In this work, three different magnetite-based ferrofluids have been synthesized by co-precipitation. A double-fatty acid layer of OA, LA, and MA renders the aqueous solutions stable and biocompatible. The characterization of the samples shows similar inorganic cores around 10 nm, with large size distributions, which is noticeable for MA@NP. The surface characterization confirmed the fatty acids’ presence, whose extents are different for the MA@NP sample. The magnetic characterization showed superparamagnetic particles with saturation values close to 80 Am^2^/kg. The assessment of δM and Henkel plots showed the dominant presence of demagnetizing interactions for the three samples, but especially for MA@NP, probably again because of larger particles within the sample.

We tested these samples in three different bioapplications. They all showed promising results for magnetic hyperthermia and relaxation measurements. The $r_{2}$ values of our samples largely exceed those of commercial Endorem^®^, and their ILP values were also satisfactory.

The clustered LA@NP are excellent for detection labeling, as concluded from the application of the studied particles for lateral flow immunoassays and their magnetic quantification. These nanolabels are superparamagnetic at room temperature, which provides a high initial magnetic susceptibility and contributes to excellent colloidal stability. The fatty acid’s carboxylic groups can be activated permitting the amide bond for conjugation to the protein. The LA@NP form monodisperse-sized clusters gathering a few particles, which increases the magnetic material per unit molecule without hindering the regular flowing or the antigen–antibody reactions. The fatty acid coating also enabled a smooth flow along the LFA membrane that presented no detectable residuals out of the test and control lines and the fiber pads.

## Figures and Tables

**Table 1:** Mean particle diameter $d_{\mathrm{TEM}}$ by TEM and its standard deviation *σ*; average crystallite size $d_{\mathrm{XRD}}$ and its uncertainty, magnetic diameter $d_{\mathrm{MAG}}$ from the Langevin equation and its standard deviation *σ*, hydrodynamic diameter $d_{\mathrm{DLS}}$, and its polydispersity index (PDI) and ζ-potential.

Sample	$d_{TEM}$		$d_{XRD}$		$d_{MAG}$		$d_{DLS}$		ζ-Potential
	(nm)	*σ*	(nm)	±	(nm)	*σ*	(nm)	PDI	(mV)
OA@NP	10.3	0.3	6.7	0.9	9.1	0.4	120	0.120	–50
LA@NP	7.9	0.2	5.8	0.6	7.6	0.3	23	0.169	−47
MA@NP	9.5	0.3	5.8	0.9	8.9	0.4	99	0.176	−50

**Table 2:** Saturation magnetization at 250 K ($M_{S}{}^{250K}$), saturation magnetization at 5 K ($M_{S}{}^{5K}$), coercive field at 5 K ($\mu_{0}H_{C}{}^{5K}$), reduced remanence ($M_{S}/M_{R}{}^{5K}$), effective anisotropy constant (K), and real and imaginary components of the initial magnetic susceptibility, χ′ and χ′, respectively. Uncertainties in the last digits are given in parenthesis.

Sample	$M_{S}{}^{250K}\;({\mathrm{Am}}^{2}/\mathrm{kg})$	$M_{S}{}^{5K}\;({\mathrm{Am}}^{2}/\mathrm{kg})$	$\mu_{0}H_{C}{}^{5K}\;(T)$	$M_{S}/M_{R}{}^{5K}$	$K\;(J/m^{3})$	χ′	χ″
OA@NP	71(1)	79(1)	0.024	0.37	2.2(2)·10^4^	21.03	0.01
LA@NP	73(1)	80(1)	0.026	0.41	2.1(2)·10^4^	15.88	0.03
MA@NP	67(1)	74(1)	0.016	0.25	1.9(2)·10^4^	10.14	0.02

**Table 3:** Longitudinal ($r_{1}$) and transverse ($r_{2}$ ) relaxivities of fatty acid MNPs at given fields, H = 0.2 T, 0.5 T, and 1.33 T and for comparison of Endorem^®^, withdrawn commercial product.

Sample	$r_{1}\;@0.2T$ (s^−1^·mmol^−1^·L)	$r_{1}\;@0.5T$ (s^−1^·mmol^−1^·L)	$r_{1}\;@1.33T$ (s^−1^·mmol^−1^·L)	$r_{2}\;@0.2T$ (s^−1^·mmol^−1^·L)	$r_{2}\;@0.5T$ (s^−1^·mmol^−1^·L)	$r_{2}\;@1.33T$ (s^−1^·mmol^−1^·L)
OA@NP	31.0	16.2	4.8	210.0	207.7	220.1
LA@NP	24.2	11.1	3.9	195.9	197.4	251.6
MA@NP	36.5	19.4	5.5	244.3	253.3	263.5
Endorem^®^	32.9	24.0	12.3	124.9	125.0	131.6

**Table 4:** Neutravidin concentrations for the particle bioconjugation and the corresponding main parameters of the two-peaked volume distribution of sizes.

	Neutravidin Concentration	Peak 1	Peak 2	Peak 1 Volume Fraction (%)	Peak 2 Volume Fraction (%)
	(mg/mL)	$d_{DLS}\;(\mathrm{nm})$	$d_{DLS}\;(\mathrm{nm})$		
OA@NP	0	45	158	34	66
	0.75	59	208	25	75
	1	58	446	11	89
	2	107	553	9	91
LA@NP	0	23	0	100	0
	0.75	36	141	58	42
	1	38	149	45	55
	2	41	175	50	50
MA@NP	0	46	141	44	56
	0.75	105	442	26	74
	1	118	540	14	86
	2	53	212	15	85

## Data Availability

Not applicable.

## Supplementary Materials

The following are available online at https://www.mdpi.com/article/10.3390/nano12020205/s1. Figure S1: XRD patterns for the three samples OA@NP, LA@NP, and MA@NP compared with the standard Fe_3_O_4_. The value for the mean crystallite size $d_{\mathrm{XRD}}$ has been estimated from the Rietveld refinement of the XRD patterns; Figure S2: TGA curves and their first derivative of the three samples (a) OA@NP, (b) LA@NP and (c) MA@NP. The presence of a double peak in the derivative of the curves suggests the presence of a double layer of surfactant on the surface of the particles. The first peak suggests the release and decomposition of slightly bound or physically adsorbed surfactant molecules. On the other hand, the second weight loss that took place at higher temperatures could be due to the breaking of stronger bonds, i.e., chemically bound surfactants molecules to the surface of the particles, and their decomposition. Inset of the graphs show the whole TGA thermogram from 25 to 950 °C; Figure S3: From bottom to top, FTIR spectrum comparison of the OA@NP and OA, LA@NP and LA, and MA@NP and MA.; Figure S4: Intensity (black), number (red), and volume (blue) distributions for the sample LA@NP functionalized with 0.75 mg/mL of neutravidin.

## References

[1] Sung H., Ferlay J., Siegel R.L., Laversanne M., Soerjomataram I., Jemal A., Bray F. (2021). Global Cancer Statistics 2020: GLOBOCAN Estimates of Incidence and Mortality Worldwide for 36 Cancers in 185 Countries. CA Cancer J. Clin..

[2] Hamilton W. (2020). Cancer diagnostic delay in the COVID-19 era: What happens next?. Lancet Oncol..

[3] Blay J.Y., Boucher S., Le Vu B., Cropet C., Chabaud S., Perol D., Barranger E., Campone M., Conroy T., Coutant C. (2021). Delayed care for patients with newly diagnosed cancer due to COVID-19 and estimated impact on cancer mortality in France. ESMO Open.

[4] Quesada-González D., Merkoçi A. (2018). Nanomaterial-based devices for point-of-care diagnostic applications. Chem. Soc. Rev..

[5] Chun P. (2009). Colloidal Gold and Other Labels for Lateral Flow Immunoassays. Lateral Flow Immunoassay.

[6] Estelrich J., Sánchez-Martín M.J., Busquets M.A. (2015). Nanoparticles in magnetic resonance imaging: From simple to dual contrast agents. Int. J. Nanomed..

[7] Rogosnitzky M., Branch S. (2016). Gadolinium-based contrast agent toxicity: A review of known and proposed mechanisms. BioMetals.

[8] Chang D., Lim M., Goos J.A.C.M., Qiao R., Ng Y.Y., Mansfeld F.M., Jackson M., Davis T.P., Kavallaris M. (2018). Biologically Targeted Magnetic Hyperthermia: Potential and Limitations. Front. Pharmacol..

[9] Marchianò V., Salvador M., Moyano A., Gutiérrez G., Matos M., Yáñez-Vilar S., Piñeiro Y., Rivas J., Martínez-García J.C., Peddis D. (2021). Electrodecoration and Characterization of Superparamagnetic Iron Oxide Nanoparticles with Bioactive Synergistic Nanocopper: Magnetic Hyperthermia-Induced Ionic Release for Anti-Biofilm Action. Antibiotics.

[10] Salvador M., Martínez-García J.C., Fernández-García M.P., Blanco-López M.C., Rivas M. (2021). Biological and Medical Applications of Magnetic Nanoparticles. Magnetic Measurement Techniques for Materials Characterization.

[11] Moyano A., Serrano-Pertierra E., Salvador M., Martínez-García J.C., Rivas M., Blanco-López M.C. (2020). Magnetic lateral flow immunoassays. Diagnostics.

[12] Quesada-González D., Merkoçi A. (2015). Nanoparticle-based lateral flow biosensors. Biosens. Bioelectron..

[13] Panferov V.G., Safenkova I.V., Zherdev A.V., Dzantiev B.B. (2017). Setting up the cut-off level of a sensitive barcode lateral flow assay with magnetic nanoparticles. Talanta.

[14] Huang Z., Hu S., Xiong Y., Wei H., Xu H., Duan H., Lai W. (2019). Application and Development of Superparamagnetic Nanoparticles in Sample Pretreatment and Immunochromatographic Assay. TrAC-Trends Anal. Chem..

[15] Lam T., Pouliot P., Avti P.K., Lesage F., Kakkar A.K. (2013). Superparamagnetic iron oxide based nanoprobes for imaging and theranostics. Adv. Colloid Interface Sci..

[16] Stephen Z.R., Kievit F.M., Zhang M. (2011). Magnetite nanoparticles for medical MR imaging. Mater. Today.

[17] Ortega D., Pankhurst Q.A., O’Brien P. (2012). Magnetic Hyperthermia.

[18] Moyano A., Salvador M., Martínez-García J.C., Socoliuc V., Vékás L., Peddis D., Alvarez M.A., Fernández M., Rivas M., Blanco-López M.C. (2019). Magnetic immunochromatographic test for histamine detection in wine. Anal. Bioanal. Chem..

[19] Moyano A., Serrano-Pertierra E., Duque J.M., Ramos V., Teruel-Barandiarán E., Fernández-Sánchez M.T., Salvador M., Martínez-García J.C., Sánchez L., García-Flórez L. (2021). Magnetic Lateral Flow Immunoassay for Small Extracellular Vesicles Quantification: Application to Colorectal Cancer Biomarker Detection. Sensors.

[20] Bica D., Vékás L., Avdeev M.V., Marinicǎ O., Socoliuc V., Bǎlǎsoiu M., Garamus V.M. (2007). Sterically stabilized water based magnetic fluids: Synthesis, structure and properties. J. Magn. Magn. Mater..

[21] Lago-Cachón D., Oliveira-Rodríguez M., Rivas M., Blanco-López M.C., Martínez-García J.C., Moyano A., Salvador M., García J.A. (2017). Scanning Magneto-Inductive Sensor for Quantitative Assay of Prostate-Specific Antigen. IEEE Magnetics Letters.

[22] Muscas G., Singh G., Glomm W.R., Mathieu R., Kumar P.A., Concas G., Agostinelli E., Peddis D. (2015). Tuning the size and shape of oxide nanoparticles by controlling oxygen content in the reaction environment: Morphological analysis by aspect maps. Chem. Mater..

[23] Avdeev M.V., Mucha B., Lamszus K., Vékás L., Garamus V.M., Feoktystov A.V., Marinica O., Turcu R., Willumeit R. (2010). Structure and in vitro biological testing of water-based ferrofluids stabilized by monocarboxylic acids. Langmuir.

[24] Borchert H., Shevchenko E.V., Robert A., Mekis I., Kornowski A., Grübel G., Weller H. (2005). Determination of nanocrystal sizes: A comparison of TEM, SAXS, and XRD studies of highly monodisperse CoPt_3_ particles. Langmuir.

[25] Chantrell R.W., Popplewell J., Charles S.W. (1978). Measurements of particle size distribution parameters in ferrofluids. IEEE Trans. Magn..

[26] Unni M., Uhl A.M., Savliwala S., Savitzky B.H., Dhavalikar R., Garraud N., Arnold D.P., Kourkoutis L.F., Andrew J.S., Rinaldi C. (2017). Thermal Decomposition Synthesis of Iron Oxide Nanoparticles with Diminished Magnetic Dead Layer by Controlled Addition of Oxygen. ACS Nano.

[27] Clogston J.D., Patri A.K. (2011). Zeta potential measurement. Methods Mol. Biol..

[28] Wang Y., Xu H., Wei M., Gu H., Xu Q., Zhu W. (2009). Study of superparamagnetic nanoparticles as labels in the quantitative lateral flow immunoassay. Mater. Sci. Eng. C.

[29] Niculaes D., Lak A., Anyfantis G.C., Marras S., Laslett O., Avugadda S.K., Cassani M., Serantes D., Hovorka O., Chantrell R. (2017). Asymmetric Assembling of Iron Oxide Nanocubes for Improving Magnetic Hyperthermia Performance. ACS Nano.

[30] Lan Q., Liu C., Yang F., Liu S., Xu J., Sun D. (2007). Synthesis of bilayer oleic acid-coated Fe_3_O_4_ nanoparticles and their application in pH-responsive Pickering emulsions. J. Colloid Interface Sci..

[31] Lenin R., Joy P.A. (2016). Role of Primary and Secondary Surfactant Layers on the Thermal Conductivity of Lauric Acid Coated Magnetite Nanofluids. J. Phys. Chem. C.

[32] Chen M.J., Shen H., Li X., Ruan J., Yuan W.Q. (2016). Magnetic fluids’ stability improved by oleic acid bilayer-coated structure via one-pot synthesis. Chem. Pap..

[33] Zhang L., He R., Gu H.C. (2006). Oleic acid coating on the monodisperse magnetite nanoparticles. Appl. Surf. Sci..

[34] Li W., Zaloga J., Ding Y., Liu Y., Janko C., Pischetsrieder M., Alexiou C., Boccaccini A.R. (2016). Facile preparation of multifunctional superparamagnetic PHBV microspheres containing SPIONs for biomedical applications. Sci. Rep..

[35] Yang K., Peng H., Wen Y., Li N. (2010). Re-examination of characteristic FTIR spectrum of secondary layer in bilayer oleic acid-coated Fe_3_O_4_ nanoparticles. Appl. Surf. Sci..

[36] Avdeev M.V., Bica D., Vekas L., Aksenov V.L., Feoktystov A.V., Rosta L., Garamus V.M., Willumeit R. (2009). Structural aspects of stabilization of magnetic fluids by mono-carboxylic acids. Proceedings of the Solid State Phenomena.

[37] Bloemen M., Brullot W., Luong T.T., Geukens N., Gils A., Verbiest T. (2012). Improved functionalization of oleic acid-coated iron oxide nanoparticles for biomedical applications. J. Nanoparticle Res..

[38] Zhang H., Zeng D., Liu Z. (2010). The law of approach to saturation in ferromagnets originating from the magnetocrystalline anisotropy. J. Magn. Magn. Mater..

[39] Cornell R.M., Schwertmann U. (2003). The Iron Oxides.

[40] Livesey K.L., Ruta S., Anderson N.R., Baldomir D., Chantrell R.W., Serantes D. (2018). Beyond the blocking model to fit nanoparticle ZFC/FC magnetisation curves. Sci. Rep..

[41] Wohlfarth E.P. (2004). Relations between Different Modes of Acquisition of the Remanent Magnetization of Ferromagnetic Particles. J. Appl. Phys..

[42] Atkinson W.J., Brezovich I.A., Chakraborty D.P. (1984). Usable Frequencies in Hyperthermia with Thermal Seeds. IEEE Trans. Biomed. Eng..

[43] Lartigue L., Innocenti C., Kalaivani T., Awwad A., Sanchez Duque M.D.M., Guari Y., Larionova J., Gueírin C., Montero J.L.G., Barragan-Montero V. (2011). Water-dispersible sugar-coated iron oxide nanoparticles. An evaluation of their relaxometric and magnetic hyperthermia properties. J. Am. Chem. Soc..

[44] Lasoialfari A., Filibian M., Sangregorio C., Carretta P. (2013). In vivo biomedical applications of magnetic resonance and magnetic materials. Riv. Del. Nuovo Cim..

[45] Haase C., Nowak U. (2012). Role of dipole-dipole interactions for hyperthermia heating of magnetic nanoparticle ensembles. Phys. Rev. B Condens. Matter Mater. Phys..

[46] Andreu I., Natividad E., Solozábal L., Roubeau O. (2015). Nano-objects for addressing the control of nanoparticle arrangement and performance in magnetic hyperthermia. ACS Nano.

[47] Engelmann U., Buhl E.M., Baumann M., Schmitz-Rode T., Slabu I. (2017). Agglomeration of magnetic nanoparticles and its effects on magnetic hyperthermia. Curr. Dir. Biomed. Eng..

[48] Kruk D., Korpała A., Taheri S.M., Kozłowski A., Förster S., Rössler E.A. (2014). ^1^H relaxation enhancement induced by nanoparticles in solutions: Influence of magnetic properties and diffusion. J. Chem. Phys..

[49] Basini M., Guerrini A., Cobianchi M., Orsini F., Bettega D., Avolio M., Innocenti C., Sangregorio C., Lascialfari A., Arosio P. (2019). Tailoring the magnetic core of organic-coated iron oxides nanoparticles to influence their contrast efficiency for Magnetic Resonance Imaging. J. Alloys Compd..

[50] Lago-Cachón D., Rivas M., Martínez-García J.C., Oliveira-Rodríguez M., Blanco-López M.C., García J.A. (2017). High frequency lateral flow affinity assay using superparamagnetic nanoparticles. J. Magn. Magn. Mater..

[51] Salvador M., Gallo-Cordova Á., Moyano A., Martínez-García J.C., Blanco-López M.C., Puerto Morales M., Rivas M. (2020). Improved magnetic lateral flow assays with optimized nanotags for point-of-use inductive biosensing. Analyst.

[52] Sai N., Chen Y., Liu N., Yu G., Su P., Feng Y., Zhou Z., Liu X., Zhou H., Gao Z. (2010). A sensitive immunoassay based on direct hapten coated format and biotin–streptavidin system for the detection of chloramphenicol. Talanta.

[53] Serebrennikova K.V., Samsonova J.V., Osipov A.P. (2018). Enhancement of the Sensitivity of a Lateral Flow Immunoassay by Using the Biotin–Streptavidin System. Moscow Univ. Chem. Bull..

[54] Stetefeld J., McKenna S.A., Patel T.R. (2016). Dynamic light scattering: A practical guide and applications in biomedical sciences. Biophys. Rev..

