# Supplementary material for: Double-Layer Fatty Acid Nanoparticles as a Multiplatform for Diagnostics and Therapy
Source: Nanomaterials (Basel). 2022 Jan 8;12(2):205. doi: 10.3390/nano12020205 (PMC8780348; doi:10.3390/nano12020205)
Supplement: Supplementary file 1 [file nanomaterials-12-00205-s001.zip › nanomaterials-1499583-supplementary.pdf]

nanomaterials

Supplementary Material

Double-Layer Fatty Acid Nanoparticles as a Multiplatform for Diagnostics and Therapy

María Salvador [1,2], José Luis Marqués-Fernández [1], José Carlos Martínez-García [1], Dino Fiorani [2], Paolo Arosio [3], Matteo Avolio [4], Francesca Brero [4], Florica Balanean [5], Andrea Guerrini [6], Claudio Sangregorio [6,7], Vlad Socoliuc [5], Ladislau Vekas [5], Davide Peddis [2,8,*] and Montserrat Rivas [1,*]

1 Department of Physics and IUTA, Campus de Viesques, University of Oviedo, 33203 Gijón, Spain; salvadormaria@uniovi.es (M.S.); UO254204@uniovi.es (J.L.M.-F.); jcmg@uniovi.es (J.C.M.-G.)
2 Institute of Structure of Matter—National Research Council (CNR), 00015 Monterotondo Scalo, Rome, Italy; Dino.Fiorani@ism.cnr.it
3 Department of Physics, Università degli studi di Milano and INFN, 20133 Milano, Italy; paolo.arosio@unimi.it
4 Department of Physics, Università degli studi di Pavia and INFN, 27100 Pavia, Italy; matteo.avolio01@universitadipavia.it (M.A.); francesca.brero@unipv.it (F.B.)
5 Romanian Academy—Timisoara Branch, Center for Fundamental and Advanced Technical Research, Laboratory for Magnetic Fluids, 300222 Timisoara, Romania; floricabalanean@gmail.com (F.B.); vsocoliuc@gmail.com (V.S.); vekas.ladislau@gmail.com (L.V.)
6 INSTM and Dipartimento di Chimica "Ugo Schiff", Università di Firenze, 50019 Sesto Fiorentino, Italy; andrea.guerrini@sns.it (A.G.); csangregorio@iccom.cnr.it (C.S.)
7 ICCOM-CNR and INSTM, 50019 Sesto Fiorentino, Italy
8 Department of Chemistry and Industrial Chemistry, Università degli Studi di Genova, 16146 Genova, Italy
* Correspondence: davide.peddis@unige.it (D.P.); rivas@uniovi.es (M.R.)

S1. X-ray Diffraction

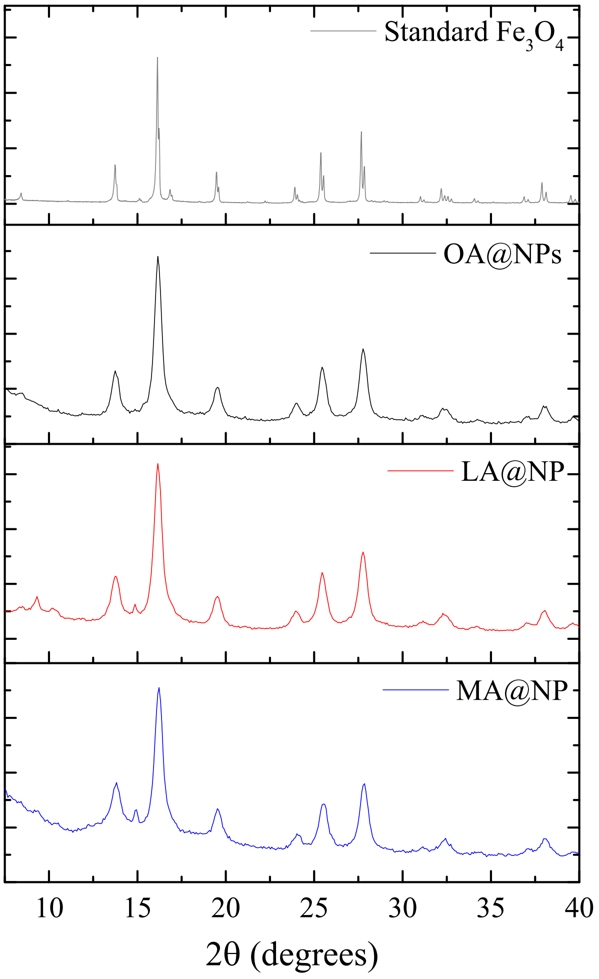

Figure S1. XRD patterns for the three samples OA@NP, LA@NP, and MA@NP compared with the standard Fe_3O_4. The value for the mean crystallite size d_{XRD} has been estimated from the Rietveld refinement of the XRD patterns.

S2. TGA Curves

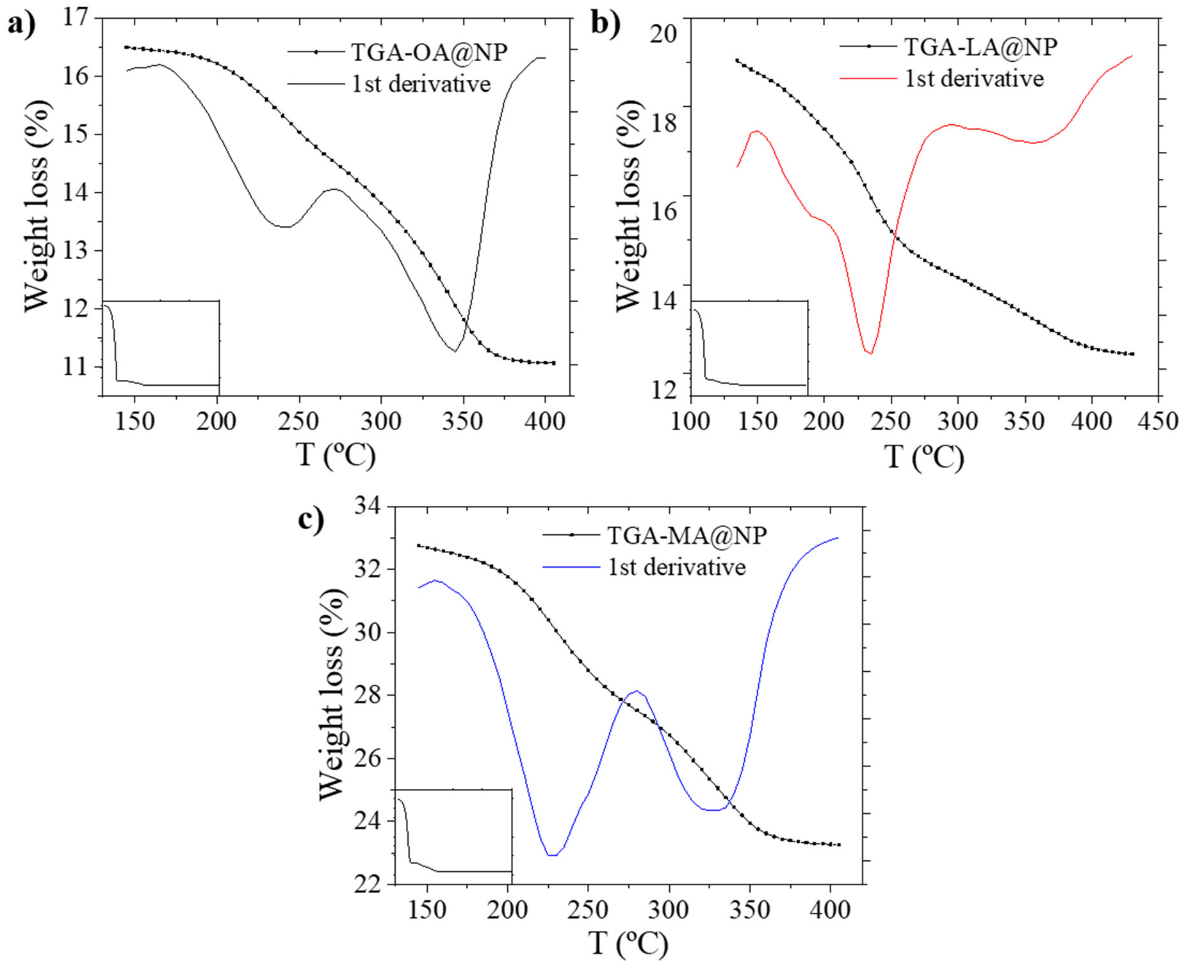

Figure S2. TGA curves and their first derivative of the three samples (**a**) OA@NP, (**b**) LA@NP and (**c**) MA@NP. The presence of a double peak in the derivative of the curves suggests the presence of a double layer of surfactant on the surface of the particles. The first peak suggests the release and decomposition of slightly bound or physically adsorbed surfactant molecules. On the other hand, the second weigh loss that took place at higher temperatures could be due to the breaking of stronger bonds, i.e. chemically bound surfactants molecules to the surface of the particles, and their decomposition. Inset of the graphs show the whole TGA thermogram from 25 to 950 °C.

S3. FTIR Spectra

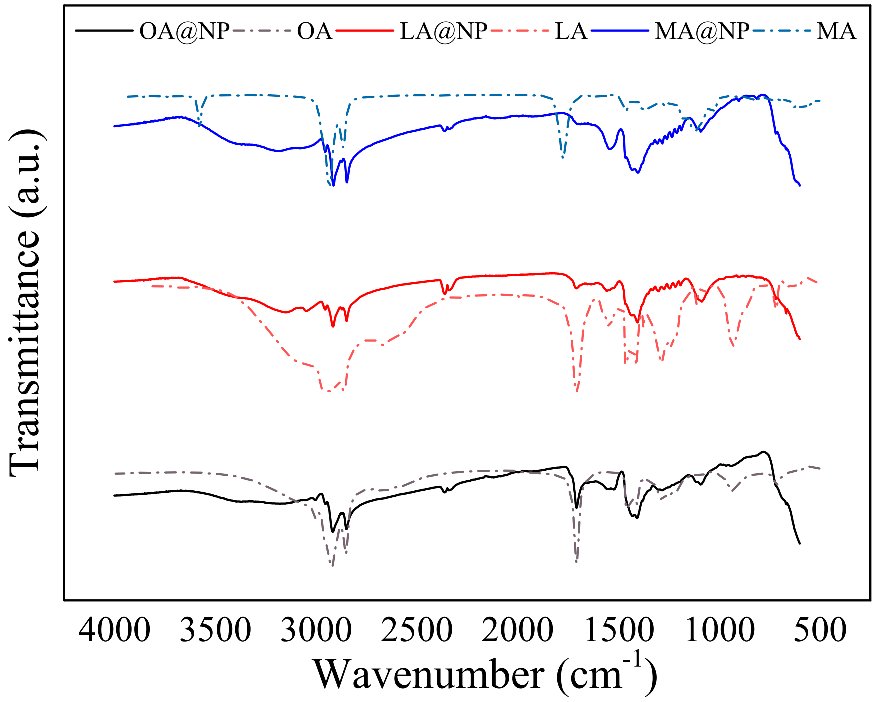

Figure S3. From bottom to top, FTIR spectrum comparison of the OA@NP and OA, LA@NP and LA and MA@NP and MA.

S4. IRM and DCD Remanence Curves

The field dependence of remanent magnetization was measured using the IRM (Isothermal Remanent Magnetization) and DCD (Direct Current Demagnetization) protocols. According to the IRM protocol, the samples, in the demagnetized state, were cooled in a zero magnetic field down to 5 K. At this temperature, a small external field was applied for 10 s, then switched off, and finally, the remanence (m^IRM) was measured. The process was repeated, increasing the field in steps up to 5 T. In a DCD measurement, the initial state was the magnetically saturated one. After cooling the sample at 5 K, an external field of -5 T was applied for 10 s, then it is turned off and the remanence (m^DCD) was measured. As in IRM, a small external field in the opposite direction to magnetization was applied for 10 s and then switched off. Finally, the remanent magnetization was measured. This was repeated increasing the field up to +5 T.

S5. Biofunctionalization Process

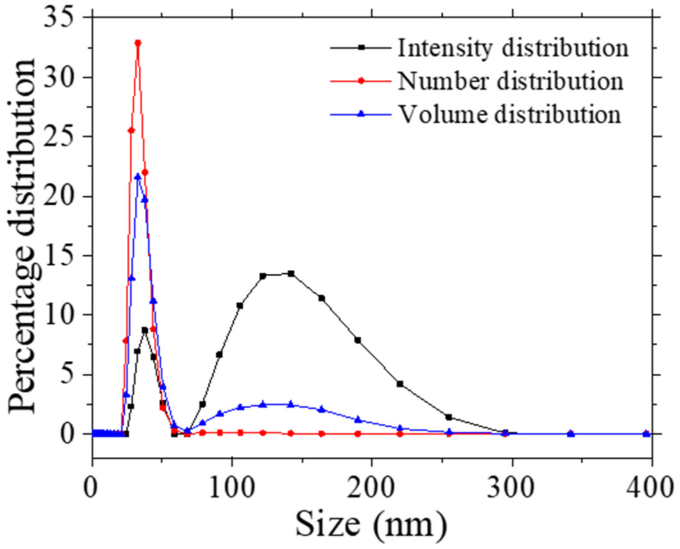

Figure S4. Intensity (black), number (red) and volume (blue) distributions for the sample LA@NP functionalized with 0.75 mg/mL of neutravidin.
